# Blinded Independent Central Review (BICR) in New Therapeutic Lung Cancer Trials

**DOI:** 10.3390/cancers13184533

**Published:** 2021-09-09

**Authors:** Hubert Beaumont, Antoine Iannessi, Yi Wang, Charles M. Voyton, Jennifer Cillario, Yan Liu

**Affiliations:** 1 Median Technologies, 1800 Route des Crêtes, 06560 Valbonne, France; hubert.beaumont@mediantechnologies.com (H.B.); antoine.iannessi@mediantechnologies.com (A.I.); yi.wang@mediantechnologies.com (Y.W.); charles.voyton@mediantechnologies.com (C.M.V.); jennifer.cillario@mediantechnologies.com (J.C.); 2 Centre Antoine Lacassagne, 33 Avenue de Valombrose, 06100 Nice, France

**Keywords:** lung cancer, clinical trial, RECIST 1.1, blinded independent central review

## Abstract

**Simple Summary:**

Lung cancer treatment has dramatically evolved in the past decade, but some pitfalls of image interpretation have been introduced in parallel, such as pseudo-progressions. These challenges could be made more evident with blinded independent central reviews, as readers are often blinded to patient clinical symptoms and outcomes. The aim of this study was to analyze a pool of lung trials that used RECIST 1.1, document the proportion of reader discrepancies and the reader performance through monitoring procedures, and provide suggestions for the reduction of read inconsistency. This study provides benchmarks for the reader discordance rate in novel lung cancer therapeutic trials that will help to trigger corrective actions such as initial reader training and follow-up re-training.

**Abstract:**

Background: Double reads in blinded independent central reviews (BICRs) are recommended to control the quality of trials but they are prone to discordances. We analyzed inter-reader discordances in a pool of lung cancer trials using RECIST 1.1. Methods: We analyzed six lung cancer BICR trials that included 1833 patients (10,684 time points) involving 17 radiologists. We analyzed the rate of discrepancy of each trial at the time-point and patient levels as well as testing inter-trial differences. The analysis of adjudication made it possible to compute the readers’ endorsement rates, the root causes of adjudications, and the proportions of “errors” versus “medically justifiable differences”. Results: The trials had significantly different discrepancy rates both at the time-point (average = 34.3%) and patient (average = 59.2%) levels. When considering only discrepancies for progressive disease, homogeneous discrepancy rates were found with an average of 32.9%, while readers’ endorsement rates ranged between 27.7% and 77.8%. Major causes of adjudication were different per trial, with medically justifiable differences being the most common, triggering 74.2% of total adjudications. Conclusions: We provide baseline performances for monitoring reader performance in trials with double reads. Intelligent reading system implementation along with appropriate reader training and monitoring are solutions that could mitigate a large portion of the commonly encountered reading errors.

## 1. Introduction

In the past decade, the lung cancer treatment landscape has dramatically evolved, increasingly branching out thanks to better understanding of disease mechanisms of action, novel technologies, and some amount of serendipity in drug development. The previous perception of cancer as a distinct organ-specific disease is now largely replaced by one involving smaller distinct entities, each responding to different biological pathways, paving the way to treatments that specifically target cancer-specific mutational genotypes [1]. The trend in targeted treatments has been led by epidermal growth factor receptor (EGFR) inhibitors, closely followed by the anaplastic lymphoma kinase (ALK) inhibitors [2]. More recently, cancer immunotherapy has pushed this revolution to a new peak, thanks to the remarkable improvements in patient overall survival attained with immune checkpoint agents [3]. Today, approximately 2500 clinical trials (719 phase I studies, 975 phase II studies, 288 phase III studies, 29 phase IV studies, and 380 studies for which a phase stage is not applicable) registered on clinicaltrial.gov (https://clinicaltrials.gov, accessed on 10 August 2021) are about to recruit or are actively recruiting in order to investigate new therapeutics of lung cancer, offering new hope to patients for better survival and for improvements in quality of life.

In pivotal lung cancer trials, overall survival remains a preferred endpoint in assessing drug efficacy; however, overall survival exhibits disadvantages in its inability to account for post-trial life-prolonging therapy, which is far from being standardized [4]. In addition, tracking overall survival can also be time- and cost-intensive. Surrogate endpoints, including progression free survival (PFS) and objective response rate (ORR), derived using the new Response Evaluation Criteria in Solid Tumors (RECIST 1.1) [5] are commonly used in lung cancer. The acceptance of the use of these surrogate endpoints by regulatory authorities has allowed for more rapid drug development, which has in turn increased patient access to cutting-edge drugs that help combat these deadly diseases.

Blinded independent central reviews (BICRs) are advocated in clinical trials to independently verify endpoints and control bias that might result from errors in response or progression assessments. In the BICR settings with double reads, the medical images are reviewed by two independent readers blinded to the results of the other reader, the study treatment, the investigator assessment, and some pre-defined clinical information. The double-reading paradigm creates the possibility for discordance between the two readers; therefore, a third radiologist is involved to make the final decision of the evaluation outcome [6]. The monitoring of reader performance is required by regulatory bodies to ensure data quality and reliability. At the trial level, a high adjudication rate could be an alert of poor quality at the study level, and a low number of endorsements from a given reader would raise concerns about the reliability of that specific reader [7]. Therefore, relevant key performance indicators (KPIs) must be designed and implemented before starting the reads; these allow the study monitor to trigger corrective actions accordingly. A pooled analysis of 79 oncology clinical trials by Ford et al. [7] showed that the proportion of cases requiring adjudication among the 11 lung cancer trials included in the analysis was 38% (95% CI: 37–40%) [7]. However, this study was general to all cancer types and did not included details on discrepancy root cause or recently approved novel therapeutics. Considering the atypical response patterns provided by those drugs [8,9], we thought it prudent to provide an update on reader performance specific to new therapeutics in lung cancer.

Focusing on BICRs in assessing novel drugs, the aim of this study was to analyze a pool of lung trials using RECIST 1.1, document the proportion of reader discrepancies, and provide suggestions to aid in improving the read consistency of future trials by estimating relevant KPIs.

## 2. Materials and Methods

### 2.1. Study Data Inclusion Criteria

This study (ClinicalTrials.gov identifier NCT05038826) included the BICRs from six clinical trials (trials 1–6), including immunotherapy and targeted therapy, examining lung cancer and performed between 2017 and 2021. The selected BICR trials were conducted with double reads with adjudication, and assessments were based on RECIST 1.1 guidelines (Table 1). All data were blinded with respect to the study sponsor, study protocol number, therapeutic agent under study, and subject demographics and identifying information. For these six trials, a total of 1821 patients were expected, totaling 17 radiologists (Rad 1–17) and involving 7 adjudicators. The central reads were all performed using the same radiological reading platform (LMS; Median Technologies, France).

### 2.2. Read Paradigm

**Figure 1:**
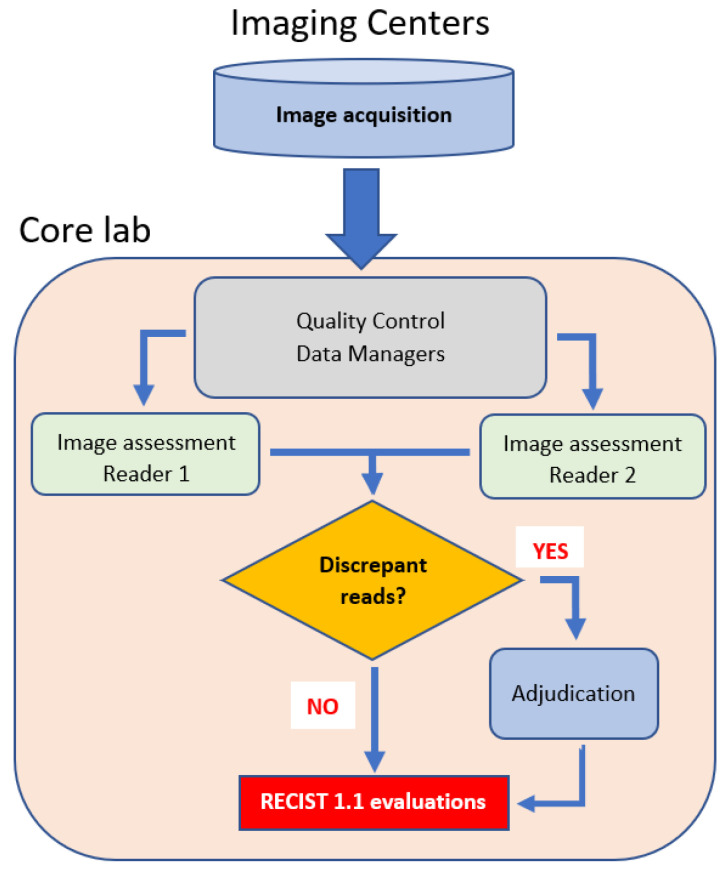
Workflow of the BICR with double reads and adjudication.

Two independent radiologists performed the review of each image and determined the radiologic time-point response (RTPR) in accordance with RECIST 1.1. According to the trials’ endpoints (response or progression), specific types of discrepancies triggered adjudications that were pre-defined in an imaging review charter (Figure 1). The adjudicator (a third independent radiologist) reviewed the response assessments from the two primary readers and endorsed the outcome of one of the readers, providing a rationale to endorse the adjudicators’ assessments. Finally, a medical lead investigated the discrepancies and the outcomes of all adjudications to monitor the study.

### 2.3. Reader Variability Monitoring

Assessments of read discordance are part of the quality program that tracks any inherent reader variability. Monitoring processes usually rely on several read performance KPIs, including the inter-reader discordance rate, the adjudication rate, the endorsement rate, and the error rate used to identify reader outliers. The adjudicator and the medical monitor document every discrepancy event along with the possible root causes, which here included four RECIST-derived categories along with two operationally based causes (Table 2 and Table 3). These discordances were also categorized according to the type of expected discordance: “read error” or “medically justifiable difference”. Regarding individual reader performances, we provide average values for each reader individually, as well as together with an estimate of the lowest acceptable endorsement rate. To ensure a meaningful statistical analysis, radiologists who were involved in less than 25 adjudications were excluded from this study.

### 2.4. Analysis Plan and Statistics

First, we analyzed the rate of discrepancy for all trials using the following considerations:

- Considering discordances at any time point, independently of patient chronology. This KPI consists of the sum of all discrepant TPs out of the sum of all TPs for a given trial;
- Considering that at least one discordance occurred when reading the patient follow-up. This KPI is the same as the KPI described above but is taken at the patient level;
- Considering only discrepancies in the date of progression (DOP) and the adjudication rate based on this endpoint. This KPI allows meaningful comparison between trials.

For these analyses, we computed average trial performances and tested for inter-trial differences in their discrepancy rates. We also tested for significant correlation with the average number of time points per patient.

Second, we analyzed readers’ performances in all trials for the:

- Reader endorsement rate;
- Proportion of “errors” and “medically justifiable differences”;

For these analyses, we documented the root causes of discrepancies from the adjudicator and medical monitor opinions.

The discrepancy rate of each trial was computed using a Clopper–Pearson model for the computation of exact confidence intervals of proportions. The Marascuilo test for multiple proportions [10] was used for comparing intra- and inter-trial proportions. Across trials, we computed the Pearson correlation between the average number of time point per patient and the discrepancy rate. From previously computed distributions of discrepancy and endorsement rates across trials, we derived warning limits aiming at early detection of underperforming trials. We computed the minimum sample size required to reliably detect those trials. We performed sample size estimation for a one-sample proportion test with a 5% level of significance at 80% power. We estimated a one-sided significant difference between (1) the average trial discrepancy rate and a 50% limit value and (2) between the average endorsement rate (50%) and a 25% limit value. R Cran software was used and the significance level was 5%.

## 3. Results

### 3.1. Trial Monitoring

For each trial all discordance rates, considering any kind of discordance, are summarized in Table 2. A pairwise comparison showed that, at the patient level, discordance rates were significantly different between trial 3 and trial 5. At the time-point level, the discordance rates of trial 3 and trial 5 were significantly different compared to the other four trials (1, 2, 4, and 6). We found a significant correlation between the discordance rate and the average number of time points per patient, at the patient level (*p* = 0.049) and at time-point level (*p* = 0.034). Table 2 further shows the discordance rates when discordance was restricted to DOP, which had an overall value of 32.9% [30.7; 35.1]. Pairwise trial–comparisons showed no significantly different discordance rates among all trials. However, we found a significant (*p* = 0.05) Pearson correlation of 0.72 between the average number of TPs per patient (see Table 1) and trials’ discordance rates based on DOP.

**Figure 2:**
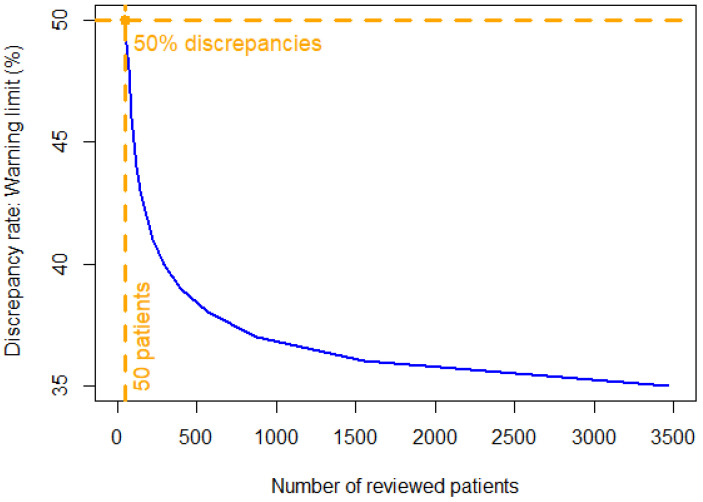
Warning limit for monitoring the discrepancy rate in the DOP. Limit of acceptable discrepancy rate measured as a function of the number of patients read in the trial. In orange: a discrepancy rate higher than 50% after reviewing more than 50 patients warns of suboptimal performances in the trial.

From the average value of 32.9% shown in Table 2 for the DOP-based discordance rate, we derived warning limits for detecting underperforming trials. Figure 2 shows the warning limits computed to detect underperforming trials according to the number of patients assessed in the trial. In a running trial for which more than 50 patients have already been evaluated by the two readers, a DOP-based discrepancy rate higher than 50% would be an early KPI to inform about trial quality.

**Figure 3:**
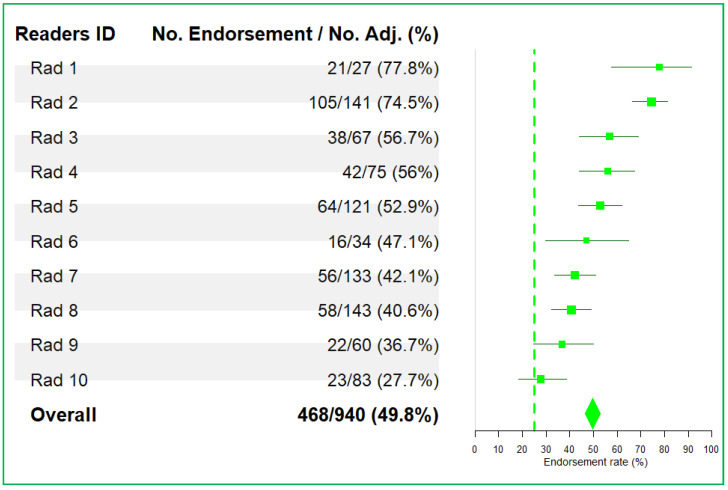
Reader endorsement rates after adjudication: Adjudication was based on discrepancy in the DOP. The dashed green line at 25% indicates a significant difference from the average reader endorsement (50%) for readers involved in more than 25 adjudications.

For the analysis of individual reader performance, we summarized the endorsement rates of 10 readers who were involved in more than 25 adjudications (Figure 3). The average reader endorsement rates were near 50%, ranging from 27.7% to 77.8% across readers.

### 3.2. Root Causes of Adjudicated Discrepancies

As the final stage of our analysis, we aggregated all root causes for adjudications per trial, according to RECIST-derived root causes assigned by adjudicators. Table 3 reports the distribution of the root causes responsible for triggering adjudications. Predefined root causes are listed in columns, and adjudication paradigms were trial-specific. For each clinical trial and each root cause, the numbers of occurrences and corresponding percentages (in parenthesis) are reported (Table 3).

In averaging all trial adjudications, the proportions due to lesion selection, new lesion detection, and the measurement of lesions were not significantly different. However, the predominant causes of adjudication were significantly different for trials 1 and 6 for lesion measurement, trial 2, 3 and 4 for the detection of new lesions, and trial 5 for the selection of lesions.

In Table 4, we report, for each trial, the proportion of “read errors” and “medically justifiable differences”. We found that, when pooling the six trials, 74.2% (95% CI: [71.2; 77.0]) of adjudications were deemed “justifiable differences”. Taken separately, all trials had higher proportions of “justifiable differences” than “errors”.

## 4. Discussion

We analyzed the discordance rates and reader performances of six clinical trials examining lung cancer. Considering any kind of discrepancy in RTPR, the rates differed across trials, ranging between 15.8% and 43.4% with an average of 34.3%. Considering the patient level, at least one RTPR was discrepant on average for 59.2% of patients, and the rates also differed across trials, ranging from 32.7% to 69.5%. Per trial, discrepancy rates were significantly correlated to the average numbers of time points per patient. When considering the DOP as the single discrepancy variable, the average rate decreased to 32.9% (95% CI: [30.7; 35.1]), and no significant differences were found between trials, even if we found, per trial, a borderline correlation between the discordance rate and the average numbers of time points per patient. A low rate of endorsement within the reader pool was a warning signal to trigger investigation specifically into one reader. The endorsement rate ranged from 27.7% to 77.8%. Using these results, we computed warning limits as KPIs able to detect suboptimal trials early. These warning limits are applicable to specific trials involving targeted therapy and immunotherapy and adaptable to different study sizes.

Three trials featured the detection of new lesions as a major reason for discordance, triggering more than 40% of total adjudications. We previously reported a similar conclusion in a phase II SCLC trial [11] where 57.3% of adjudications were attributable to new lesions, which highlights a limitation of RECIST in the evaluation of lymph nodes. However, we observed that the nature and frequency of adjudication root causes varied across trials: for two trials, the major cause was the measurement of target lesions and in another it was the selection of lesions. Depending on the disease and the treatment [12], it can be difficult to judge lesion etiology, especially without clinical and biological patient information as an independent reader. In light of this, we further separated the adjudications into two sub-classes: “read errors” and “medically justifiable differences”. This study shows that 74.2% of the total adjudications were medically justifiable.

**Figure 4:**
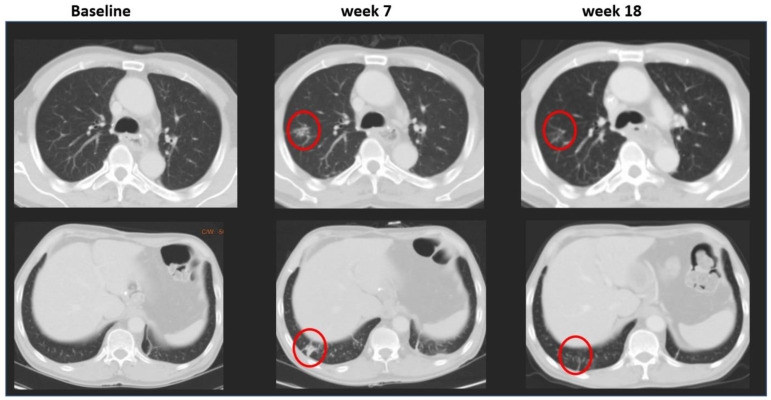
New micro- or ground-glass lesions. Multiple new lung nodules marked in red circles (partial solid or ground-glass opacities) appeared in week 7, were determined as equivocal in the same week, and resolved in week 18.

New appearance of lymph nodes is a typical example of the types of challenges encountered in lung cancer trials [11]. It is important to remember that to meet the criteria for malignancy, lymph nodes must meet the size threshold (≥10 mm) to be considered new lesions. Discordance may occur due to measurement variations; for example, a 9 mm lymph node at baseline that increases to 11 mm at a follow-up visit. It has also been reported that a sarcoid-like reaction that manifests with mediastinal and hilar lymphadenopathy in patients treated with immunotherapy serves as a common source of discordance [12]. In such situations, the RECIST 1.1 working group recommended that, when a single pathologic node is driving the progression event, continuation of treatment with confirmation by a subsequent exam should be strongly considered [13]. Another typical finding that represents another challenge in lung cancer is the new appearance of single or multiple micronodules, or ground-glass opacities (Figure 4), possibly due to immune-related adverse events, which can be interpreted as new lesions triggering progression. In such cases, follow-up images are critical to verify whether the lesion size has significantly increased and truly represents progression of lung cancer.

**Figure 5:**
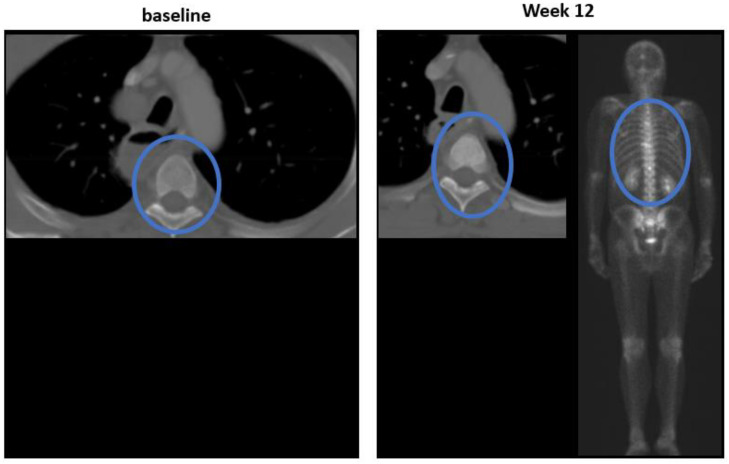
New sclerotic lesions. Newly appearing sclerotic lesions in the CT and uptakes in the bone scan in week 12, but with no evidence of lesions at baseline (marked in blue circles).

About 30–40% of patients with lung cancer develop bone metastases during the course of their disease [14]; blastic lesions are also problematic, as they often represent a positive treatment effect, as opposed to progressive disease in bone (Figure 5). Therefore, new sclerotic lesions in the CT (that may correspond to new areas of increased tracer uptake on bone scans) should be re-evaluated to see if there is an occult lytic lesion present in the CT at baseline. In such cases, it is likely that the new sclerotic lesion represents a positive treatment effect. Evaluation of sclerotic lesions for progression should take into consideration the length of time of the therapy, as well as the status of the target lesions and non-target lesions and the likelihood of the occurrence of the flare phenomena.

**Figure 6:**
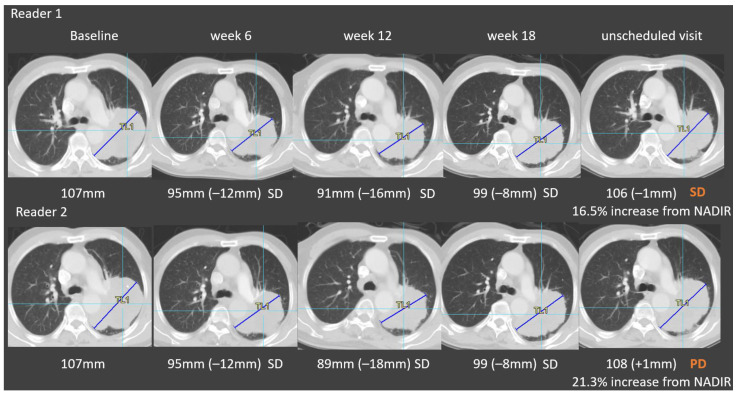
Variability in target lesion measurements. Reader 1 and reader 2 selected the same target lesion at baseline. Even though the two readers performed very similar subsequent measurements, at the third visit, a small measurement difference triggered discrepant responses. At the final visit, one reader declared a stable disease (SD) whereas the other reader declared a progressive disease (PD).

To assess response, the reliability of the target lesion selection and measurement is essential [15]; with regard to errors in target selection, our analysis revealed that a proportion of target lesions were selected in previously irradiated areas. As recommended by RECIST 1.1, previously treated lesions should not be selected as target lesions unless progression has been clearly documented [5]. In this regard, prior local treatment information is critical for BICR target lesion selection, and it should be considered as a read error if such clinical information is not considered; reader retraining serves as an effective solution. For target lesion measurement (Figure 6), variability in measurements can be controlled to a certain extent; for instance, by standardizing the reviewing process or using software tools [16].

Our study has several limitations. First, the investigation of the medical monitors was based on the adjudicators’ assessments of read errors and medically justifiable cases, which could have been limited by the variable quality of the adjudicators’ comments, and this prevented rigorous statistical processing of the data. Second, even though it can be hypothesized that, due to their different mechanisms of action, treatments (and their combinations [17]) can have an impact on tumor evaluations, this study did not analyze the treatment impact as a co-variable of discordance. In the data we assumed that the two trial arms were blinded, so we were blinded from the treatment and were only able to analyze the discrepancies derived from multiple treatments pooled together. Third, the generalizability of our results could also be a concern as these trials were all performed using the same image analysis platform. The LMS^®^ reading platform features a set of automatic controls, aimed at checking the conformance to RECIST 1.1 (e.g., max number of target lesions, max number of target lesions per organ) and the follow-up integrity (e.g., pairing of tumors), and an electronic case report form. Therefore, the automatic checks also avoided some of the errors that would have happened otherwise.

## 5. Conclusions

In BICR trials with double reads, adjudication rates can fluctuate drastically depending on the selected adjudication paradigm, the average numbers of time points per patient, and other covariates, such as the challenges of image interpretation related to novel lung cancer therapies. Our analysis shows that trial monitoring requires cross-reference analysis for the definition of baseline KPI values. A model of the expected DOP discordance rate can be built to estimate the data reliability, and it should be implemented with caution in the similar contexts of image reading and clinical trial indication. At the reader level, the endorsement rate is another valuable KPI for monitoring. These metrics help to trigger corrective actions, such as initial reader training and follow-up re-training. Group training is useful to discuss challenging cases and reach consensus to reduce discrepancies.

## Figures and Tables

**Table 1:** Description of included trials. Primary study endpoints were: progression-free survival (PFS) and overall response rate (ORR). Most patients were treated for non-small cell lung cancer (NSCLC).

Trials ID	Indication	Phase	Expected Numbers of Patients	Therapy	Primary Study Endpoints
Trial 1	NSCLC	III	340	Immune checkpoints + chemotherapy vs. chemotherapy + placebo	PFS
Trial 2	NSCLC	III	389	Immune checkpoints + chemotherapy vs. chemotherapy + placebo	PFS
Trial 3	SCLC	II	100	RNA-polymerase-II inhibitor	ORR
Trial 4	NSCLC	III	266	Tyrosine kinases inhibitor	PFS
Trial 5	NSCLC	II	366	Tyrosine kinases inhibitor	ORR
Trial 6	NSCLC	III	360	Immune checkpoints + chemotherapy vs. chemotherapy + placebo	PFS

**Table 2:** Discordance rates per trial.

Trial ID	No. of TPs Reviewed	TP Level Discordance Rate (%)	No. of Patients	Patient Level (All Types) Discordance Rate (%)	Patient Level (DOP Only) Discordance Rate (%)	Average TP/Patient
Trial 1	1570	29.9 [26.9; 31.5]	327	59.0 [53.4; 64.4]	33.0 [27.9; 38.4]	4.8
Trial 2	1386	30.0 [27.6; 32.5]	360	56.2 [50.8; 61.3]	33.9 [29.0; 39.0]	3.8
Trial 3	290	15.8 [11.8; 20.6]	107	32.7 [23.9; 42.4]	25.2 [17.3; 34.5]	2.7
Trial 4	2610	34.9 [33.1; 36.8]	278	61.8 [55.9; 67.6]	39.9 [34.1; 45.9]	9.4
Trial 5	2706	43.4 [41.5; 45.3]	357	69.5 [64.4; 74.2]	30.5 [25.8; 35.6]	7.6
Trial 6	2122	30.7 [28.7; 32.7]	404	58.2 [53.2; 63.0]	31.2 [26.7; 35.9]	5.2
Total	10,684	34.3 [33.4; 35.2]	1833	59.2 [56.9; 61.4]	32.9 [30.7; 35.1]	5.8

From left to right: number of time points (TPs) reviewed in the trial; rate of any RTPR discordance at the study level (independently of the study patients belonged to); number of patients included in the trial; rates of patient discordance with at least one RTPR discordant at any TP; rate of patient discordance (DOP only); average number of TPs per patient in the trial. When applicable, the corresponding 95% CI is shown in brackets.

**Table 3:** Distribution of the causes of adjudication. Raw numbers and proportions relative to the total numbers in the trials (%). Adjudications were documented by adjudicators, and adjudication paradigms are trial-specific.

Trial ID	Lesion Selection	New Lesion Detection	Non-Target Lesion PD	Lesion Measurement	Missing Data	Image Quality	Sum
Trial 1	58 (30.8%)	38 (20.2%)	10 (5.3%)	82 (43.6%)	0 (0%)	0 (0%)	188
Trial 2	11 (14.8%)	38 (51.4%)	8 (10.8%)	17 (23.0%)	0 (0%)	0 (0%)	74
Trial 3	5 (14.7%)	14 (41.2%)	2 (5.9%)	13 (38.2%)	0 (0%)	0 (0%)	34
Trial 4	46 (25.6%)	80 (44.5%)	12 (6.7%)	40 (22.3%)	1 (0.6%)	1 (0.6%)	180
Trial 5	128 (51%)	57 (22.7%)	12 (4.8%)	54 (21.5%)	0 (0%)	0 (0%)	251
Trial 6	34 (20.6%)	30 (18.2%)	14 (8.5%)	86 (52.1%)	0 (0%)	1 (0.6%)	165
Sum (N)	282 (31.6%)	257 (28.8%)	58 (6.6%)	292 (32.7%)	1 (0.1%)	2 (0.2%)	892

**Table 4:** Distribution of the types of adjudications.

Trial ID	Errors	Justifiable Differences	Sum
Trial 1	28	160	188
Trial 2	8	66	74
Trial 3	4	30	34
Trial 4	60	120	180
Trial 5	100	151	251
Trial 6	30	135	165
Sum (N)	230 (25.8%)	662 (74.2%)	892

## Data Availability

Data sharing is not applicable to this article.

## Abbreviation

BICR	Blinded independent central review
CAD	Computer-aided detection
CT	Computed tomography
DOP	Date of progression
KPI	Key performance indicator
LMS	Lesion management solution
NSCLC	Non-small cell lung cancer
ORR	Overall response rate
OS	Overall survival
PD	Progressive disease
PFS	Progression-free survival
RECIST	Response evaluation criteria in solid tumors
RTPR	Radiologic time-point response
SCLC	Small-cell lung cancer
SD	Stable disease
